# A recombinase polymerase amplification assay for rapid detection of rabies virus

**DOI:** 10.1038/s41598-021-82479-8

**Published:** 2021-02-04

**Authors:** Martin Faye, Ahmed Abd El Wahed, Oumar Faye, Jonas Kissenkötter, Bernd Hoffmann, Amadou Alpha Sall, Ousmane Faye

**Affiliations:** 1 Virology Department, Institut Pasteur de Dakar, 36, Avenue Pasteur, 220 Dakar, Senegal; 2 Virology Lab, Division of Microbiology and Animal Hygiene, University of Göttingen, Göttingen, Germany; 3 Institute of Animal Hygiene and Veterinary Public Health, University of Leipzig, Leipzig, Germany; 4 Institute of Diagnostic Virology, Friedrich-Loeffler-Institute, Greifswald-Insel Riems, Germany

**Keywords:** Developmental biology, Molecular biology, Diseases

## Abstract

Rabies is a generally fatal encephalitis caused by a negative-sense single-stranded RNA lyssavirus transmitted to humans mainly from dog bite. Despite the recommendation by WHO and OIE to use the direct immunofluorescence test as standard method, molecular diagnostic assays like reverse transcription quantitative polymerase chain reaction (RT-qPCR) are increasing as a confirmatory method. However, both technologies are inaccessible in resource-limited settings. Moreover, the available point-of-need molecular assay is of poor detection limit for African strains. Herein, we developed a reverse transcription recombinase polymerase amplification (RT-RPA) assay as potential point-of-need diagnostic tool for rapid detection of various strains of rabies virus including locally isolated African strains. The sensitivity and specificity of the method was evaluated using a molecular RNA standard and different Rabies-related viruses belonging to the *Rhabdoviridea* family, respectively. The RABV-RPA performances were evaluated on isolates representative of the existing diversity and viral dilutions spiked in non-neural clinical specimen. The results were compared with RT-qPCR as a gold standard. The RABV-RPA detected down to 4 RNA molecules per reaction in 95% of the cases in less than 10 min. The RABV-RPA assay is highly specific as various RABV isolates were identified, but no amplification was observed for other member of the *Rhabdoviridea family*. The sample background did not affect the performance of the RABV-RPA as down to 11 RNA molecules were identified, which is similar to the RT-qPCR results. Our developed assay is suitable for use in low-resource settings as a promising alternative tool for *ante-mortem* rabies diagnosis in humans for facilitating timely control decisions.

## Introduction

Rabies virus (RABV) is an enveloped pathogen belonging to the *Lyssavirus* genus (order Mononegavirales, family *Rhabdoviridae*) and its genome is a negative single-stranded RNA of approximately 12 kilobases (kb) in size encoding five proteins (N, P, M, G, L) separated by four intergenic regions and encompassed between a leader region and a trailer (3′ and 5′ non-coding regions)^1^. The RABV presents a large genetic diversity worldwide, with particularly four phylogenetic groups circulating in Africa^2,3^. It is responsible of a neglected zoonotic disease affecting several mammals in many parts of the world and humans are generally exposed through dog bites^4,5^. However, Rabies transmission was also reported through transplantation of organs from undiagnosed donors with a long incubation period of the virus^6,7^. Rabies infection is fatal in most of cases with an annual death toll of 60,000 in humans worldwide^1^.

The highest public health impact of Rabies is recorded in Asian and African developing countries^4,8^ and 40% of deaths occur in children under 15 years-old^5,9^. Nevertheless, early detection of cases is crucial to provide rapid outbreak response and emergency vaccination measures. Several methods have been previously described for the diagnosis of rabies virus (RABV) infection including direct virus isolation^10^, immunohistochemistry^10–12^, immunochromatography^13,14^ and immunofluorescence^15,16^, as well as sensitive molecular methods such as real-time reverse transcription polymerase chain reaction (RT-PCR) assay^17–22^. However, development of reliable broad spectrum and economical tests remains a challenge to improve surveillance of rabies cases in the field to achieve goal #3 of the sustainable development goals^17,20,23^.

Recombinase polymerase amplification (RPA) is an isothermal molecular tool with portable instrumentation currently used in many diagnostic fields as an alternative to the PCR^24,25^. RPA is becoming a molecular tool of choice for the rapid, specific, and cost-effective identification of pathogens. RPA is integrated in point-of-care (POC) bioassays, suitcase lab and on handheld automated fluidic platforms^26,27^. The method rely on a bacterial recombinase enzyme to anneal primers to template and isothermal DNA polymerase for the DNA amplification step. A reverse transcription RPA (RT-RPA) platform utilized both reverse Transcriptase Moloney Murine Leukemia Virus for the RT step and a fluorescent probe system for real-time data acquisition^28–30^. In the past decade, RPA technology was widely used for molecular diagnosis of pathogens of public health concern^31–38^. Because of its simplicity (few and easy hands-on steps), flexibility (large range of commercial kits available in dried formats), completely isothermal (i.e. no need to perform an enzyme pre-activation) and low-temperature profile (37–42 °C) and speed (results in 5–20 min), RPA technology has been successfully used for rapid detection of various pathogens without requirement of any sophisticated equipment^25,39^. RT-RPA for the rapid detection of RABV was previously developed (Schlottau assay)^21^, but we have discovered that it is not suitable for African RABV strains.

In this study, a rapid and sensitive fluorescent probe-based RT-RPA assay was developed and evaluated for rapid and broad range detection of Rabies virus (RABV). Limit of detection was measured with a synthetized standard RNA. Diagnostic accuracy was determined with collection of positive and negative specimen from various virus strains. Finally, clinical performance was checked using cerebro-spinal fluid spiked with RABV.

## Results

### Primers selection

A total of 4 forward primers (FPs), 4 reverse primers (RPs), and one exo probe targeting the conserved region of the nucleocapsid gene (N) were initially designed. The 16 combinations were screened with the 10^5^ RNA molecules/reaction of the in vitro transcribed RNA standard. The combination with the highest and earliest start of exponential amplification curve was selected and tested with tenfold serial dilutions (from 10^5^ to 10 RNA molecules/reaction) of the standard RNA. Unfortunately, the selected primers pair (FP2/RP1) enabled detection down to 10^3^ RNA molecules/reaction, which was not enough. Therefore, many primers were designed in order to select the one that produced the most sensitive assay. In total, 179 additional combination were screened. The primer pair (RPA_N_FP2C/RPA_N_RP4C3P) enabled detection down to 10 molecule/reaction of the molecular RNA standard.

### Analytical sensitivity

**Figure 1:**
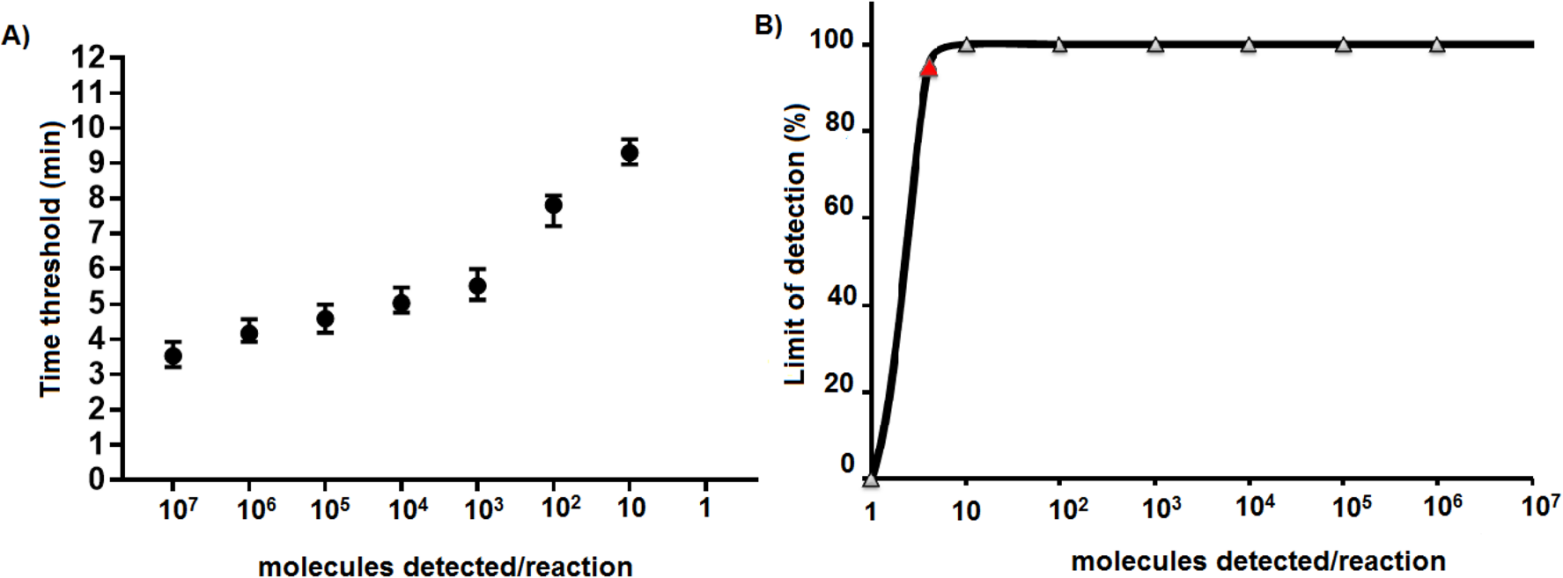
Analytical sensitivity of the RABV-RPA assay. A semi-log regression analysis (**A**) was performed by plotting the RABV-RPA threshold time values against the number of RNA molecules detected in eight replicates (8/8). The RABV-RPA assay produced positive results between 3 and 10 min. Dilutions 10^7^ to 10 RNA molecules were detected 8 out of 8 runs and 1 molecule was not identified by the RT-RPA assay. The dots represent the mean values and the error bars represent the standard deviation. The probit regression analysis (**B**) was performed using data of eight RABV-RPA assay runs. The limit of detection at 95% probability (4 RNA molecules/reaction) is depicted by the red triangle. The graphs were plotted using PRISM (Graphpad Software Inc., San Diego, California).

The analytical sensitivity was determined with Threshold time (Tt) data values from eight sets of tenfold dilutions of the molecular standard RNA ranging from 10^7^ to 1 molecules/reaction. As low as 10 molecules/reaction within 10 min was detected in the new RABV RT-RPA (Fig. 1A). The probit analysis revealed the limit of detection in 95% of cases was 4 RNA molecules/reaction (Fig. 1B). Intra-run and inter-run coefficients of variation (CVs) were calculated from eight Tt data values of the dilution 10^5^ standard RNA molecules/reaction tested in the same run and in eight different runs. Intra-run and inter-run (CVs) were 0.54% and 3.40% for the RABV-RPA assay, respectively.

### Analytical specificity

All RABV strains were detected while no cross-reaction was observed with rabies-related virus species belonging to the *Rhadoviridae* family, showing that the RABV-RPA assay is highly specific for RABV detection (Table 1). To confirm the specificity of the developed RT-RPA assay, five RPA amplicons from RABV isolates belonging to distant clades were purified and sequenced using the Illumina Miseq platform (Illumina, San Diego, CA, USA). The BLAST analysis (https://blast.ncbi.nlm.nih.gov/) of obtained sequences showed an identity ranging from 98.5 to 100% to the target region of related sequences available in GenBank. Assembled sequences from the RPA amplicons were deposited in GenBank under the accession numbers MW123050-54.

**Table 1:** Description and results of rhabdoviruses isolates used in this study for specificity assessment.

Isolates	Virus	Genus	Reference	Place of isolation	Year of isolation	Species	Rabies virus RT-qPCR		RABV-RPA	
							Mean Cq value^a^	SD	Mean Tt value^b^	SD
SA221203SEN	Rabies (RABV)	*Lyssavirus*	NRC-rabies IPD	Senegal	2011	*Mellivora capensi*	24.07	0.18	2.53	0.01
SA217694SEN	Rabies (RABV)	*Lyssavirus*	NRC-rabies IPD	Senegal	2011	*Canis lupus familiaris*	22.41	0.28	5.97	0.12
SA217695SEN	Rabies (RABV)	*Lyssavirus*	NRC-rabies IPD	Senegal	2011	*Canis lupus familiaris*	27.44	0.07	4.81	0.02
SA217750SEN	Rabies (RABV)	*Lyssavirus*	NRC-rabies IPD	Senegal	2011	*Canis lupus familiaris*	24.99	0.20	2.70	0.06
SH218152SEN	Rabies (RABV)	*Lyssavirus*	NRC-rabies IPD	Senegal	2011	*Homo sapiens*	18.82	0.06	2.26	0.25
SH177846SEN	Rabies (RABV)	*Lyssavirus*	NRC-rabies IPD	Senegal	2005	*Homo sapiens*	18.88	0.04	2.46	0.01
SA194858SEN	Rabies (RABV)	*Lyssavirus*	NRC-rabies IPD	Senegal	2008	*Canis lupus familiaris*	26.65	0.23	3.36	0.05
SA204014SEN	Rabies (RABV)	*Lyssavirus*	NRC-rabies IPD	Senegal	2010	*Canis lupus familiaris*	19.43	0.34	2.37	0.01
SA206776SEN	Rabies (RABV)	*Lyssavirus*	NRC-rabies IPD	Senegal	2010	*Canis lupus familiaris*	19.12	0.08	2.03	0.08
SA252888SEN	Rabies (RABV)	*Lyssavirus*	NRC-rabies IPD	Senegal	2013	*Canis lupus familiaris*	18.48	0.08	2.25	0.04
SA252913SEN	Rabies (RABV)	*Lyssavirus*	NRC-rabies IPD	Senegal	2013	*Canis lupus familiaris*	21.90	0.08	2.48	0.14
SA262037SEN	Rabies (RABV)	*Lyssavirus*	NRC-rabies IPD	Senegal	2013	*Canis lupus familiaris*	20.06	0.04	2.51	0.09
SA262503SEN	Rabies (RABV)	*Lyssavirus*	NRC-rabies IPD	Senegal	2014	*Canis lupus familiaris*	21.13	0.04	2.44	0.21
SA262518SEN	Rabies (RABV)	*Lyssavirus*	NRC-rabies IPD	Senegal	2014	*Canis lupus familiaris*	20.38	0.06	2.35	0.11
SA267115SEN	Rabies (RABV)	*Lyssavirus*	NRC-rabies IPD	Senegal	2014	*Canis lupus familiaris*	19.48	0.13	2.29	0.15
DakAnB1094	Kolongo (KOLV)	unassigned	JX276998	CAR	1970	*Euplectes afra*	Neg	Neg	Neg	Neg
AnY1307	Mokola (MOKV)	*Lyssavirus*	NC_006429	Cameroon	1973	*Crocidura spp.*	Neg	Neg	Neg	Neg
AnB373d	Sandjimba (SJAV)	unassigned	JX277024	CAR	1970	*Acrocephalus schoenobaenus*	Neg	Neg	Neg	Neg
AnB4289	Nasoule (NASV)	unassigned	JX277012	CAR	1973	*Andropadus virens*	Neg	Neg	Neg	Neg
AnD42443	Lagos Bat (LBV) 3	*Lyssavirus*	NC020807	Senegal	1985	*Eidolon helvum*	Neg	Neg	Neg	Neg
AnB672	Lagos Bat (LBV) 2	*Lyssavirus*	NRC-rabies IPD	CAR	1974	*Micropteropus pusillus*	Neg	Neg	Neg	Neg
An K 6909	Lagos Bat (LBV) 4	*Lyssavirus*	NRC-rabies IPD	Guinea	1985	*Nycteris gambiensis*	Neg	Neg	Neg	Neg
LBVNIG1956	Lagos Bat (LBV) 6	*Lyssavirus*	EF547431	Nigeria	1956	*Eidolon helvum*	Neg	Neg	Neg	Neg
DakHD763	Le Dantec (LDV)	*Ledantevirus*	AY854650	Senegal	1965	*Homo sapiens*	Neg	Neg	Neg	Neg
DakAnD5314	Keuraliba (KEUV)	*Ledantevirus*	JX276996	Senegal	1968	*Tatera kempi*	Neg	Neg	Neg	Neg
ArD89384	Chandipura (CHNV)	*Vesiculovirus*	NRC-rabies IPD	Senegal	1992	*Phlebotomus sp.*	Neg	Neg	Neg	Neg
SudAr1275	Obodhiang (OBOV)	*Ephemerovirus*	HM856902	Sudan	1963	*Mansonia uniformis*	Neg	Neg	Neg	Neg
ArY31-65	Nkolbisson (NKOV)	*Ledantevirus*	JX277015	Cameroon	1965	*Eretmapodites leucopus*	Neg	Neg	Neg	Neg
DakAnB439	Garba (GARV)	unassigned	KM204982	CAR	1970	*Corythornis cristata*	Neg	Neg	Neg	Neg
SAAr1995	Mossuril (MOSV)	*Hapavirus*	KM204993	Mozambique	1959	*Culex sitiens*	Neg	Neg	Neg	Neg

Tt: time threshold in minutes, CAR: Central African Republic, SD: standard deviation, Neg: negative; no cross-reactivity, Cq: quantitative cycle, NRC-Rabies IPD: national reference center for rabies virus (NRC-Rabies) at Institut Pasteur de Dakar, BP 220, Dakar, Senegal.

^a^Mean Cq value from duplicates with the previously described RABV-L-protein real-time RT-qPCR assay by Faye et al. used as reference test^19^.

^b^Mean Tt value from duplicates given by the new established rabies virus RT-RPA assay.

### Diagnostic performances

To ascertain the wide range detection capacity of the RABV-RPA assay in comparison to the Schlottau assay^21^ as well as the RABV-L-protein real-time RT-qPCR^19^, 24 RABV isolates from different parts of the world were tested in duplicate using the three methods^40^. In addition, a total of nineteen RABV-negative samples were also analyzed in duplicate. The RT-qPCR and the RABV-RPA assay were able to detect all positive. The median detection Tt of the RABV-RPA was 5.12 ± 1.76 min. In contrast, the Schlottau assay detected only 62.5% of analyzed isolates in a median Tt of 7.17 ± 1.62 min. The Schlottau assay gave no-fluorescence signal with nine tested isolates including those from Senegal (SA267333SEN, SA173837SEN and SH290371SEN), Burma (99009BUR), Laos (99010LAO), China (02043CHI), Brazil (86001BRE), Niger (90010NIG) and Afghanistan (02052AFG) (Table 2). All nineteen Rabies-negative samples were tested negative in all assays.

**Table 2:** Assessment of detection spectrum of the RABV-RPA assay versus real-time RT-qPCR and the Schlottau assay.

Isolates	Phylogenetic clade—subclade	Reference	Place of isolation	Year of isolation	Species	Rabies virus RT-qPCR	RABV-RPA	Schlottau assay
						Mean Cq value^a^	Mean Tt value^b^	Mean Tt value^c^
SH155966SEN	Africa 2	NRC-rabies IPD	Senegal	2001	Human	24.67	3.23	4.94
SA267333SEN	Africa 2	NRC-rabies IPD	Senegal	2014	Dog	25.36	5.58	Neg
SA173837SEN	Africa 2	NRC-rabies IPD	Senegal	2004	Dog	28.52	5.24	Neg
SH189343SEN	Africa 2	NRC-rabies IPD	Senegal	2007	Human	18.32	2.46	7.77
SA272282SEN	Africa 2	NRC-rabies IPD	Senegal	2015	Dog	18.29	1.87	5.23
SH290289SEN	Africa 2	NRC-rabies IPD	Senegal	2017	Human	22.69	2.92	7.17
SH290371SEN	Africa 2	NRC-rabies IPD	Senegal	2017	Human	23.10	5.00	Neg
91047FRA	Cosmopolitan—WE	KX148127	France	1991	Fox	20.41	6.88	6.71
96140POL	Cosmopolitan—CE	KX148120	Poland	1993	Raccoon dog	16.31	5.95	5.48
92001GER	Cosmopolitan—WE	KX148135	Germany	1991	Fox	22.68	7.32	8.14
86054YOU	Cosmopolitan—EE	KX148145	BA	1986	Wolf	25.54	7.86	10.11
87001ARS	Cosmopolitan—ME1a	NRC-rabies IPP	Saudi Arabia	1987	Fox	30.08	6.33	5.03
94009TUR	Cosmopolitan—ME2	KX148165	Turkey	1993	Dog	26.22	5.83	7.79
99009BUR	ND	NRC-rabies IPP	Burma	1999	Dog	31.12	5.97	Neg
99008CBG	Asian—SEA3	KX148252	Cambodia	1999	Dog	29.50	7.62	9.10
99010LAO	Asian—SEA3	KX148255	Laos	1999	Dog	31.61	3.77	Neg
02043CHI	Asian—SEA2a	NRC-rabies IPP	China	ND	Dog	29.60	4.02	Neg
02045CHI	Asian—SEA2a	NRC-rabies IPP	China	ND	Dog	36.11	4.02	7.59
91014MEX	Cosmopolitan—AM2a	KX148110	Mexico	1991	Dog	30.78	3.35	6.72
86001BRE	Cosmopolitan—AM3a	KX148216	Brazil	1986	Dog	28.36	4.33	Neg
90010NIG	Africa 2	KX148231	Niger	1990	Dog	27.42	7.45	Neg
91004USA	Arctic-related—A	KX148224	USA	1991	Skunk	23.64	3.93	7.25
02052AFG	Arctic-related—AL1b	KX148225	Afghanistan	2002	Dog	22.40	6.84	Neg
91041RUS	Cosmopolitan—CA1	NRC-Rabies IPP	Russia	1991	Fox	23.18	3.65	4.53

Tt: time threshold in minutes, BA: Bosnia and Herzegovina, Neg: negative; no cross-reactivity, Cq: quantitative cycle, NRC-Rabies IPD: national reference center for rabies virus (NRC-Rabies) at Institut Pasteur de Dakar, BP 220, Dakar, Senegal, NRC-Rabies IPP: national reference center for rabies virus (NRC-Rabies) at Institut Pasteur, Paris, France.

^a^Mean Cq value from duplicates with the previously described RABV-L-protein real-time RT-qPCR assay by Faye et al. used as reference test^19^.

^b^Mean Tt value from duplicates given by the RABV-RPA assay.

^c^Mean Tt value from duplicates obtained with the Schlottau assay^21^.

As the RT-qPCR used as reference test^19^, both RT-RPA assays exhibited a diagnostic specificity of 100% (95% CI 82.35–100%) and a positive predictive value (PPV) of 1. However, differences were observed in other performances. The RABV-RPA showed a diagnostic sensitivity of 100% (95% CI 85.75–100%) and a negative predictive value (NPV) of 1, while the Schlottau assay^30^ had a diagnostic sensitivity of 79.17% (95% CI 57.85–92.87%) a NPV of 0.7 (CI 95% 0.63–0.89) (Fischer’s exact test p < 0.001). A k coefficient of 1 ± 0.15 (95% CI 0.70–1.29) was determined for the RABV-RPA assay, while the Schlottau assay^21^ showed a k coefficient of 0.77 ± 0.14 (95% CI 0.47–1.06) (p < 0.05); resulting in a concordance of 100% for the RABV-RPA assay to results from the real-time RT-qPCR assay^19^ on the same samples (Table S1).

### Screening of spiked samples with the RT-RPA assays

Eleven tenfold dilutions of an archived RABV in primary cerebro-spinal fluid (CSF) were tested in duplicate with both RT-RPA assays and the real-time RT-qPCR^19^. The RT-qPCR was able to detect down to 8 RNA molecules/reaction within a mean Tt of 70.58 ± 0.120 min. However, the RABV-RPA assay detected until a titer of 11 RNA molecules/reaction in a shorter Tt of 7.49 ± 0.123 min compared to the RT-qPCR. The Schlottau assay^30^ showed detection limit of 275 RNA molecules/reaction within an average Tt of 8.97 ± 0.121 min (Table 3).

**Table 3:** Detection limit of the new established RT-RPA assay on cerebro-spinal fluid sample.

TITERS ID50/mL	RT-qPCR				RABV-RPA		Schlottauassay	
	RNA molecules/reaction	Mean Cq value^a^	Mean Tt^b^	SD	Mean Tt^b^	SD	Mean Tt^b^	SD
5.2E+05	32,033	28.42	57.68	0.480	3.10	0.020	5.33	0.030
5.2E+04	2374	31.92	61.71	0.007	4.36	0.019	7.83	0.179
5.2E+03	275	34.82	65.05	0.220	5.21	0.006	8.97	0.121
5.2E+02	82	36.45	66.92	0.672	5.45	0.086	Neg	
5.2E+01	11	39.18	70.06	0.092	7.49	0.123	Neg	
5.2	8	39.63	70.58	0.120	Neg		Neg	
5.2E−01		Neg			Neg		Neg	
5.2E−02		Neg			Neg		Neg	
5.2E−03		Neg			Neg		Neg	
5.2E−04		Neg			Neg		Neg	
5.2E−05		Neg			Neg		Neg	

Tenfold serial dilutions of RABV isolate SA217695SEN with an initial virus titer of 5.2 10^6^ ID_50_/mL were tested with an rabies virus RT-RPA assay previously described by Schlottau et al.^21^ and the new RT-RPA assay. The corresponding numbers of RNA molecules per reaction were calculated using the mean Cq values with the standard equation previously described for the RT-qPCR used here as reference technique^19^.

Neg: negative or no fluorescence signal, Cq : quantitative cycle number, Tt: time threshold (minutes).

^a^Mean Cq value from duplicates.

^b^Mean Tt value from duplicates.

Compared to the real-time RT-qPCR^19^, the sensitivity of the RABV-RPA assay using serial dilutions of a CSF sample spiked with RABV was of 83.33% (95% CI 35.88–99.58%) with an accuracy of 90.91% (95% CI 58.72–99.77%), while the Schlottau assay exhibited a clinical sensitivity of 50% (95% CI 11.81–88.19%) and an accuracy of 72.73% (95% CI 39.03–93.98%). Our RABV-RPA assay showed agreement with real-time RT-qPCR used as reference test (Cohen's Kappa test = 0.819; p = 0.005) (Table S1).

### In silico analysis of primers and probes sequences

Available coding-complete sequences from Laos (LAO), China (CHIN), Brazil (BRE), Niger (NIG), Afghanistan (AFG) and Senegal (SEN) enabled in silico evaluation of the Schlottau assay^21^ and the RABV-RPA assay using the BLAST program (https://blast.ncbi.nlm.nih.gov/).

**Figure 2:**
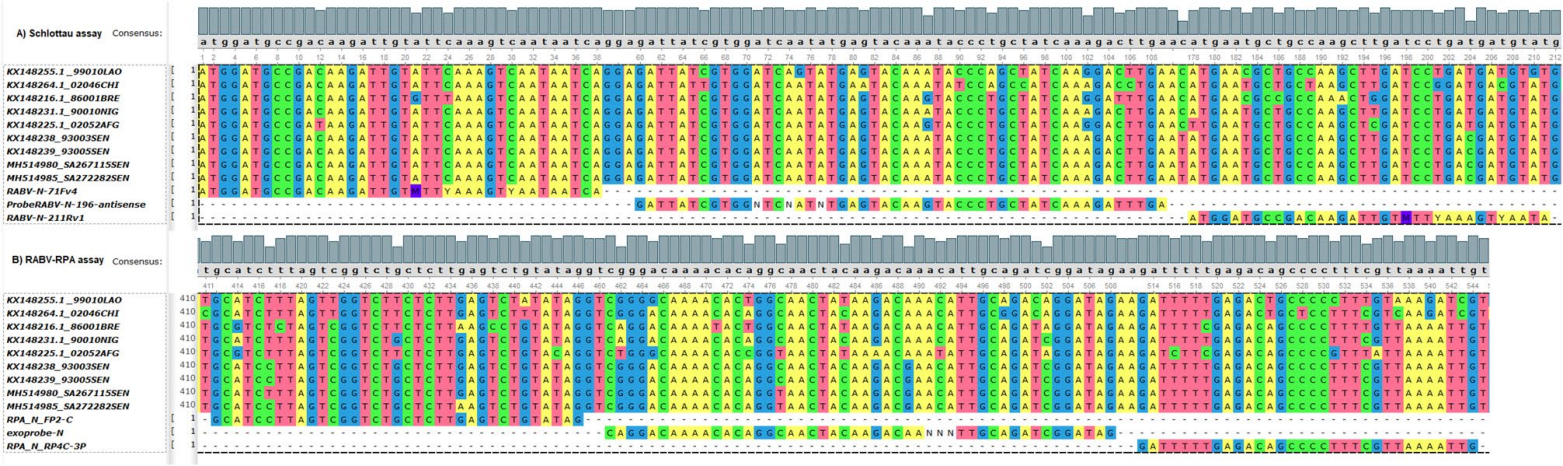
In silico analysis of (**A**) the Schlottau assay and (**B**) the RABV-RPA assay again sequences of differences rabies virus isolates from Laos (LAO), China (CHIN), Brazil (BRE), Niger (NIG), Afghanistan (AFG) and Senegal (SEN), respectively. The alignment was performed using the MAFFT algorithm implemented in the Unipro UGENE software^50^. The in silico analysis revealed signature erosion in the reverse primer of the Schlottau assay, indicating that this rabies virus diagnostic method have issues with sensitivity when used to detect some isolates from these countries.

The forward primer of the Schlottau assay (RABV-N-71Fv4) is highly similar to the target region of the isolates from Laos, China, Niger and Senegal, while it shows a dissimilarity of 5% and 2% against the isolates from Brazil and Afghanistan, respectively. The Schlottau assay’s probe (ProbeRABV-N-196-antisense) exhibits also dissimilarities of 2%, 4%, 4% and 4% to sequences from Brazil, Niger, Afghanistan and Senegal, respectively, while it is more distant to the isolates from Laos and China with dissimilarities of 10% and 16%, respectively. The reverse primer of the Schlottau assay (RABV-N-211Rv1) reveals the higher dissimilarities to undetected sequences with values ranging from 41 to 50% (Table S2; Fig. 2A).

The forward primer of the RABV-RPA (RPA_N_FP2-C) is highly similar to African sequences from Senegal and Niger with distances ranging from 0 to 3% and 3%, respectively, while it shows dissimilarities of 11% to the isolates from Laos, China, and Afghanistan. However, this primer exhibits a dissimilarity of 17% to the sequence from Brazil. The probe of the RABV-RPA (exoprobe-N) also is highly similar to the target region of the isolate from Niger and shows a dissimilarity to the Senegalese sequences between 4 and 6%. However, it is distant to the isolates from Brazil, China, Laos and Afghanistan with dissimilarities of 8%, 8%, 13% and 17%, respectively. As the corresponding forward primer, the reverse primer of the RABV-RPA is higher similar to the African sequences. However, it exhibits dissimilarities of 6%, 16%, 16% and 19% to the isolates from Brazil, China, Afghanistan and Laos, respectively (Table S2; Fig. 2B). Nevertheless, these dissimilarities didn’t omit detection of the aligned sequences by the RABV-RPA assay (Fig. 2B).

## Discussion

Despite being preventable, rabies is responsible for thousands of human deaths worldwide each year, mostly in Africa and Asia^23,41^. Many rabies endemic areas don’t have the resources to implement Direct Fluorescent Antibody testing, which also need a fluorescent microscope and highly trained technicians^16^. On the top of that, a continuous cold storage of samples is a big limiting factor. Therefore, according to the WHO orientations related to the need for rapid and economical diagnosis tests^23^, considerable efforts are made to develop accessible direct detection methods based on RT-qPCR, which allow a rapid and highly sensitive detection of RABV. Nevertheless, these technologies require the use of complex instruments and well-equipped laboratories^17–19,22^. A field-friendly assay would enable transmission control in low resource settings and help in surveillance under field condition.

In this study, we developed and validated a reliable and highly sensitive RT-RPA assay for RABV detection. The assay could be easily implemented with minimal equipment and training at remote areas, where the real burden of the disease is underestimated^41^.

Since a program or strict rules are not available, the most challenging step in the RABV-RPA assay development is the design of a primer pair able to amplify a very low RNA copy number^31,42,43^. Nevertheless, a highly sensitive RT-RPA for rapid detection of RABV was successfully established, exhibiting a reliable detection until 4 RNA molecules/reaction within 10 min. The RABV-RPA displayed only a 1-log_10_-step reduction compared to the reference standard RT-qPCR assay^19^ and is more sensitive than the Schlottau assay (1000 RNA molecules/ reaction)^21^ and a recently described RT-RPA assay (562 RNA molecules/reaction)^44^. In addition, obtaining results between 2 to 10 min demonstrated that the RABV-RPA assay is also much faster than the previously described rabies virus isothermal molecular tests^21,45–48^ without affecting the sensitivity.

Despite few mismatches, the RABV-RPA assay shows better detection performances on isolates from various regions of the world than the Schlottau assay^21^, which was less sensitive for isolates from Senegal, Burma, Laos, China, Niger, Brazil and Afghanistan. This might be related to signature erosion in it reverse primer. Therefore, our RT-RPA method is proposed as suitable for rabies diagnostic in the field, particularly in low-resource areas in West-Africa, South-Asia, South-America and Middle-East where rabies still circulating.

In contrast to the Schlottau assay, the RABV-RPA assay showed high accuracy on viral dilutions in a non-neural clinical specimen (CSF) and displayed good agreement with the RT-qPCR^19^. Based on its performances, our RABV-RPA presented here could be useful as reliable point-of-need tool for *ante-mortem* rabies diagnosis in humans, as well as a valuable surveillance tool for rapid local decision-making. Furthermore, an outstanding advantage for the RT-RPA method is that could be used with a relatively basic portable heat-source, and therefore making the RABV-RPA a potential point-of-need alternative for RABV detection in resource-limited settings. Finally, our RABV-RPA assay, could be a complement for existing methods for rabies diagnosis^19,21,22^ with a high specificity, sensitivity and repeatability and more suitable for rapid and broad detection of RABV isolates.

In conclusion, the RABV-RPA method developed in this study demonstrates high sensitivity and specificity for detection of RABV. In addition, a validation experiment showed its clinical applicability and could be used for rapid and accurate *ante-mortem* diagnosis in human. The RABV-RPA could be used in a suitcase laboratory with rapid extraction methods which had previously shown best performances in extraction of rabies virus RNA^21,31,49,50^ and easily implemented in routine laboratory activities as a rapid confirmatory test to first-line assays or in low-resource settings by including portable heat-sources with solar-powered battery^34^.

## Methods

### Ethical statement

All samples used in this study were collected in the frame of the national integrated surveillance program for Rabies in Senegal or available from routine diagnostic activities of the national reference centers for rabies diagnosis in Senegal and Paris.

According to IACUC animal guidelines^51^, suckling mice were used in routine virus isolation at the animal laboratory in Institute Pasteur in Dakar accredited by WHO as Collaborating Centre for Arboviruses and Hemorrhagic Fevers, for surveillance, diagnostics and research as approved by the Senegalese national ethical committee. All viral isolations in suckling mice were performed in accordance with the ARRIVE guidelines^52^.

### Design of RABV-RPA primers and exo probe

The RABV-RPA primers were designed by in silico multiple alignments of available Africa 1 and Africa 2 RABV sequences from GenBank using the Muscle algorithm implemented in the Unipro UGENE software^53^. According to RPA guidelines from TwistDx (Cambridge, UK), a total of seventeen forward primers (FPs), twenty seven reverse primers (RPs), and one fluorescent exo probe were designed based on the conserved region of the nucleocapsid gene (N). The exo-probe was designed with an inverse arrangement of fluorophore (6-carboxyfluorescein [FAM]), quencher (black hole quencher 1 [BHQ-1]), internal abasic site mimic (tetrahydrofuran spacer [THF]) and block elongation (phosphate [P]). All oligonucleotides were produced by TIB MolBiol (Berlin, Germany). All 179 primer combinations were tested to select the RPA primers and probe set yielding the highest analytical sensitivity using the 10^5^ RNA in vitro transcripted molecular standard ordered from Genexpress (Berlin, Germany) (Table 4).

**Table 4:** Oligonucleotide sequences of primers and probe designed for the RABV-RPA assay.

Name	Type	Length (bp)	Sense	Sequence 5′–3′	Gene	Position^a^	Product size (bp)
RPA_N_FP2C	Primer	35	Forward	GCATCCTTAGTCGGTCTGCTCTTGAGTCTGTATAG	N	482–516	133
Exo_probe_N	Probe	45	Reverse	CTATCCGATCTGCAA[BHQ-dT][THF][FAM-dT]TTGTCTTGTAGTTGCCTGTGTTTTGTCCTG–Ph	N	531–578	
RPA_N_RP4C3P	Primer	32	Reverse	CAATTTTAACGAAAGGGGCTGTCTCAAAAATC	N	583–614	

FAM: fluorescein amidit; BHQ: blackberry quencher; THF: tetrahydrofuran; Ph: phosphate; bp: base-pairs.

^a^Corresponding nucleotide positions on genome of the rabies virus strain from Nigeria DRV-NG11(GenBank Ac. No. KC196743).

### RNA extraction and samples preparation

All thirty seven virus isolates analyzed in this study were previously cultured on suckling mice brain and derived from collection of national reference center for rabies diagnosis in Senegal at Institut Pasteur de Dakar (NRC-Rabies IPD) (Tables 1, 2). A total of 17 RNA extracts from primary rabies-positive brain samples representative of the existing RABV diversity were provided by the national reference center for rabies diagnosis at Institut Pasteur of Paris, France (NRC-Rabies IPP) (Table 2). The CSF sample was obtained from the collection of the laboratory for medical analysis at IPD. Extraction was carried out in the Biosafety level 3 (BSL3) containment facilities at Institut Pasteur de Dakar respecting the WHO’s biosafety guidelines and requirements for conducting work on infectious microorganisms and other biological hazards such as RABV^54^.

Extraction of viral RNA from 140 µL of mice brain and tenfold serial dilutions of a CSF sample was performed with the QIAamp viral RNA mini kit (Qiagen, Heiden, Germany) according to manufacturer’s instructions and eluted in a final volume of 50 μL. Extracted RNA was frozen at − 80 °C prior to downstream applications.

### Rabies virus RT-RPA assay

RABV-RPA amplifications were achieved in a 50 μL volume using the TwistAmp RT exo kits (TwistDx, Cambridge, UK). For each reaction, 29.5 μl of rehydration buffer, 420 nM RPA primers, 120 nM exo probe and 6.7 µl of nuclease-free water were added into the lids. A 5 μL of viral RNA diluted 1/10 in nuclease-free water and 14 mM final concentration of magnesium acetate were subsequently added into each tube lid. Then the lids were closed carefully and the reaction mix was centrifuged into the lyophilized reaction pellet containing a dried enzyme. The mix was vortexed and spun down once again, and the tubes were immediately placed in the Twista Tubescanner device (TwistDx, Cambridge, UK) connected to a computer for real-time monitoring of fluorescence signal. The reaction was performed at 42 °C for 15 min, with brief mixing and centrifugation of reaction tubes after 230 s of the incubation in order to improve the sensitivity of the assay. Real-time detection of RT-RPA amplicons relies on the exonuclease enzyme, which cleaves at the THF site between fluorophore and quencher in the exo-probe and the generated fluorescence signal is measured each 10 s using the FAM channel. The resulting amplification curves were analyzed by Twista Studio software using a combined threshold and first derivation analysis for signal interpretation. Samples producing an exponential amplification curve above the threshold of the negative control were considered positive.

### Analytical specificity

RNA extracts from 15 RABV-positive isolates and 15 isolates of other *rhabdoviruses* genetically related to RABV were tested in duplicates in order to evaluate the specificity of the RABV-RPA assay (Table 1). Positive and negative controls containing standard RNA and nuclease-free water were included in each run. The real-time RT-qPCR assay was used as reference test and was performed using the Quantitect Probe RT-PCR Kit (Qiagen, Heiden, Germany) in a final volume of 25 μl following previously published protocol^19^. Amplicons obtained with the RABV-RPA assay from five isolates from Niger (90010NIG), Brazil (86001BRE), Afghanistan (02052AFG), Cambodia (99008CBG) and Saudi Arabia (87001ARS) (Table 2), were purified using *AmPure* magnetic beads (Agencourt Bioscience [Beckman Coulter Inc.], Beverly, MA, USA), and adapters were ligated using the Nextera XT library Prep kit according to the manufacturer’s recommendations (Illumina, San Diego, CA, USA). The purified and amplified libraries were quantified using the KAPA library quantification kit (Kapa Biosystems, Wilmington, DE, USA) and subsequently sequenced on the Illumina MiSeq platform with 76 bp paired-end reads using the Miseq reagents kit v3 according to the manufacturer’s protocol (Illumina, San Diego, CA, USA). Reads were quality trimmed using Prinseq-lite, and de novo genome assembly was performed with IDBA (Iterative de Bruijn graph De Novo Assembler). The coverage and the identity of obtained sequences to the target region were verified using a BLAST analysis (https://blast.ncbi.nlm.nih.gov/).

### Analytical sensitivity

Analytical sensitivity of the RABV-RPA assay was assessed using eight datasets from tenfold dilutions of the in vitro standard RNA produced by Genexpress (Berlin, Germany) ranging from 10^7^ to 1 molecules/reaction. The RT-qPCR was used as reference method^19^. The Tt data values are provided as the mean and standard deviation (SD). In addition, the inter-assay and intra-assay repeatability were determined by CVs from 8 Tt values of the dilution 10^5^ standard RNA molecules/reaction in 8 different runs and in the same run, respectively.

### Diagnostic performances

In order to assess its reliability and efficiency, a collection of twenty-four positive specimens from different countries of the world^40^ was tested in duplicate with both the RABV-RPA assay and the Schlottau assay^21^ as well as the real-time RT-qPCR assay as reference test^19^. Nineteen Rabies-negative samples were also used with both assays for assessment of diagnostic specificity. PPV and NPV, diagnostic sensitivity and specificity were calculated using standard formulas^19^.

### Experimental comparison of the RT-RPA assays

To assess the usefulness of the RABV-RPA assay for *ante-mortem* diagnosis of rabies in human, tenfold serial dilutions from a positive RABV stock with a concentration of 32,033 RNA molecules/reaction (titer of 5.2 10^6^ ID_50_/mL) were performed in a CSF sample tested negative for Streptococcus, Salmonella, Legionella, Leptospirosis, Borellia, Listeria, Mycoplasma and viral herpes panel (data not shown). RNA extracts were tested in duplicate with both assays in order to determine the limit of detection (LOD) in the presence of sample background. The LOD was determined in copy/ reaction using cycle quantitative (Cq) values and the standard equation from the real-time RT-qPCR assay used as reference test^19^.

### Assessment of the impact of genetic diversity on RT-RPA assays

In silico analysis of both RT-RPA assays was performed again sequences of rabies virus isolates that were not detected during the sensitivity assessment by the Schlottau assay^21^, using the MAFFT alignment algorithm implemented in the Unipro UGENE software^53^.

### Statistical methods

A semi-log regression analysis and a probit analysis were performed by plotting the RABV-RPA Tt values against the number of molecules detected in eight replicates of tenfold dilutions of the in vitro standard RNA ranging from 10^7^ to 1 molecules/reaction (8/8) using PRISM (Graphpad Software Inc., San Diego, California) and STATISTICA (StatSoft, Hamburg, Germany), respectively. We also used a kappa test to compare diagnostic performances from the RABV-RPA assay and the Schlottau assay^21^ to results of the RT-qPCR assay^19^ where the Cohen’s kappa coefficient *(k)* represents a measure of the agreement between two assays. A p-value (p) < 0.05 was considered as statistically significant. In addition, sensitivity and accuracy of both RT-RPA assays were determined with a 95% confidence interval and their concordance to the reference test was analyzed using Cohen’s kappa test and considering a p < 0.05 as significant.

## Supplementary Information

Supplementary Information.
