# Supplementary material for: A recombinase polymerase amplification assay for rapid detection of rabies virus
Source: Sci Rep. 2021 Feb 4;11:3131. doi: 10.1038/s41598-021-82479-8 (PMC7862592; doi:10.1038/s41598-021-82479-8)
Supplement: Supplementary file 1 — Supplementary Information. [file 41598_2021_82479_MOESM1_ESM.docx]

Title: A Recombinase Polymerase Amplification assay for rapid detection of rabies virus.

Martin Faye^1*^, Ahmed Abd El Wahed^2&3^, Oumar Faye^1^, Jonas Kissenkötter^2^, Bernd Hoffmann^4^, Amadou Alpha Sall^1^, Ousmane Faye^1^

1. Virology Department, Institut Pasteur de Dakar, 36, Avenue Pasteur, 220 Dakar, Senegal
2. Virology lab, Division of Microbiology and Animal hygiene, University of Goettingen, Germany
3. Institute of Animal Hygiene and Veterinary Public Health, University of Leipzig, Leipzig, Germany
4. Institute of Diagnostic Virology, Friedrich-Loeffler-Institute, Greifswald-Insel Riems, Germany

^*^Corresponding author: Dr Martin FAYE

Virology Department,

Institut Pasteur de Dakar,

36, Avenue Pasteur,

220 Dakar, Senegal,

Phone: +221.338399223

Email: martin.faye@pasteur.sn

**Supplementary material:**

Table S1: Summary of data used for the determination of assays performances.

| **Parameters** | **Assay** |  | **Positive** | **Negative** |
| --- | --- | --- | --- | --- |
| **Diagnostic performances on 24 positive and 19 negative-RABV samples** | **RT-qPCR** | **Positive** | **24** | **0** |
|  |  | **Negative** | **0** | **19** |
|  | **RABV-RPA** | **Positive** | **24** | **0** |
|  |  | **Negative** | **0** | **19** |
|  | **Schlottau assay** | **Positive** | **19** | **0** |
|  |  | **Negative** | **5** | **19** |
| **Clinical performances on 11 ten-fold serial dilutions of a RABV-positive stock in primary CSF sample** | **RT-qPCR** | **Positive** | **6** | **0** |
|  |  | **Negative** | **0** | **5** |
|  | **RABV-RPA** | **Positive** | **5** | **0** |
|  |  | **Negative** | **1** | **5** |
|  | **Schlottau assay** | **Positive** | **3** | **0** |
|  |  | **Negative** | **3** | **5** |
| The previously described RABV-L-protein real-time RT-qPCR assay by Faye et al., 2017 was used as reference test (19).  PPV, NPV, sensitivity, accuracy and specificity of both RT-RPA assays were determined with a 95% confidence interval using standard formulas (19). Their concordance to the reference RT-qPCR test (19) was analyzed using Cohen’s kappa test and considering a p <0.05 as significant. | | | | |

Table S2: Dissimilarities between primers used more distant sequences using a BLAST analysis.

| **Assay** | **Primers** | **KX148255.1 _99010LAO** | **KX148264.1_02046CHI** | **KX148216.1_86001BRE** | **KX148231.1_90010NIG** | **KX148225.1_02052AFG** | **KX148238_93003SEN** | **KX148239_93005SEN** | **MH514980_SA267115SEN** | **MH514985_SA272282SEN** |
| --- | --- | --- | --- | --- | --- | --- | --- | --- | --- | --- |
| **Schlottau assay** | **RABV-N-71Fv4** | **0%** | **0%** | **5%** | **0%** | **3%** | **0%** | **0%** | **0%** | **0%** |
|  | **ProbeRABV-N-196-antisense** | **10%** | **16%** | **2%** | **4%** | **4%** | **4%** | **4%** | **4%** | **4%** |
|  | **RABV-N-211Rv1** | **44%** | **50%** | **47%** | **41%** | **47%** | **41%** | **41%** | **41%** | **41%** |
| **RABV-RPA** | **RPA_N_FP2-C** | **11%** | **11%** | **17%** | **3%** | **11%** | **0%** | **0%** | **3%** | **3%** |
|  | **exoprobe-N** | **13%** | **8%** | **8%** | **0%** | **17%** | **4%** | **4%** | **6%** | **6%** |
|  | **RPA_N_RP4C-3P** | **19%** | **16%** | **6%** | **0%** | **16%** | **0%** | **0%** | **0%** | **0%** |
